# Metaphyseal dysplasia associated with chronic facial nerve palsy

**DOI:** 10.1007/s00381-016-3021-6

**Published:** 2016-02-04

**Authors:** Loucas Christodoulou, Efterpi Pavlidou, Cristina Spyridou, Simon Eccles, Alistair Calder, Kshitij Mankad, Maria Kinali

**Affiliations:** Pediatric Neurology Department, Chelsea and Westminister Hospital, 369 Fulham Road, London, SW109NH UK; Department of Craniofacial Surgery, Chelsea and Westminister Hospital, London, UK; Department of Radiology, Great Ormond Street Hospital, London, UK; Department of Radiology, Chelsea and Westminister Hospital, London, UK

**Keywords:** Pyle disease, Metaphyseal dysplasia, Erlenmeyer flask sign, Facial nerve palsy

## Abstract

**Introduction:**

Metaphyseal dysplasia (Pyle disease) is a rare autosomal recessive disease with impressive and characteristic radiological findings but relatively mild clinical features. It is usually incidentally diagnosed, despite the impressive radiological findings of gross metaphyseal widening and thinning of cortical bone.

**Case report:**

Herein, we report an exceptionally unusual case of metaphyseal dysplasia in association with chronic facial nerve palsy.

**Discussion:**

Chronic facial nerve palsy due to compression of the facial nerve in a patient with Pyle disease represents an unusual novelty. Furthermore, this case delineates the clinical spectrum and phenotype of such a rare clinical entity. To the best of our knowledge, this is the first time that such an association is being described.

## Introduction

Pyle disease (metaphyseal dysplasia) was first described by Edwin Pyle in 1931, as “a case of unusual bone development” [1]. Since then only few cases have been reported worldwide with a prevalence <1 per million [2]. It is usually mild and incidentally diagnosed, despite the impressive radiological findings of gross metaphyseal widening and thinning of cortical bone [3]. There is a considerable overlap with craniometaphyseal dysplasia (CMD), which is far more common and has more severe phenotypic features, including deafness and visual impairment [4].

We report an eight-year-old girl, who presented with chronic facial nerve palsy, dental malocclusion, and incidental skeletal dysplasia on imaging studies. To the best of our knowledge, this is the first time that metaphyseal dysplasia is reported in association with cranial nerve compression. The clinical and radiological evidence for and against metaphyseal dysplasia and craniometaphyseal dysplasia is considered.

## Case report

An eight-year-old girl was referred to our Pediatric Neurology service with a 6-year history of right-sided facial palsy. Her speech became acutely slurred but subsequently improved. There was no history of preceding trauma, recent infection, immunizations, or a rash. Parents declined to receive oral steroids. Brain magnetic resonance imaging (MRI) was initially obtained a year after the onset of her symptoms and was reported as normal, but it was unavailable for us to review.

At first evaluation, she was making good academic progress and she had been healthy over the last few years with normal development and physical growth. However, she had long-standing dental malocclusion for which she required extraction of five teeth.

The child was born at term by elective Caesarean section to healthy non-consanguineous parents. The mother had amniocentesis during this pregnancy as both she and father were carriers of beta thalassemia trait but without perinatal complications. She had a 16-year-old brother, who was fit and well, while family history was negative.

She had no features of facial dysmorphism such as wide nasal bridge, prominent forehead, hypertelorism, and prominent jaw. There was obvious tooth crowding with malocclusion. The rest of the neurological examination was normal. Laboratory evaluation with autoimmune and infectious screens was negative, while beta thalassemia trait was confirmed. Vitamin D deficiency was noted and replacement therapy was started.

**Fig. 1:**
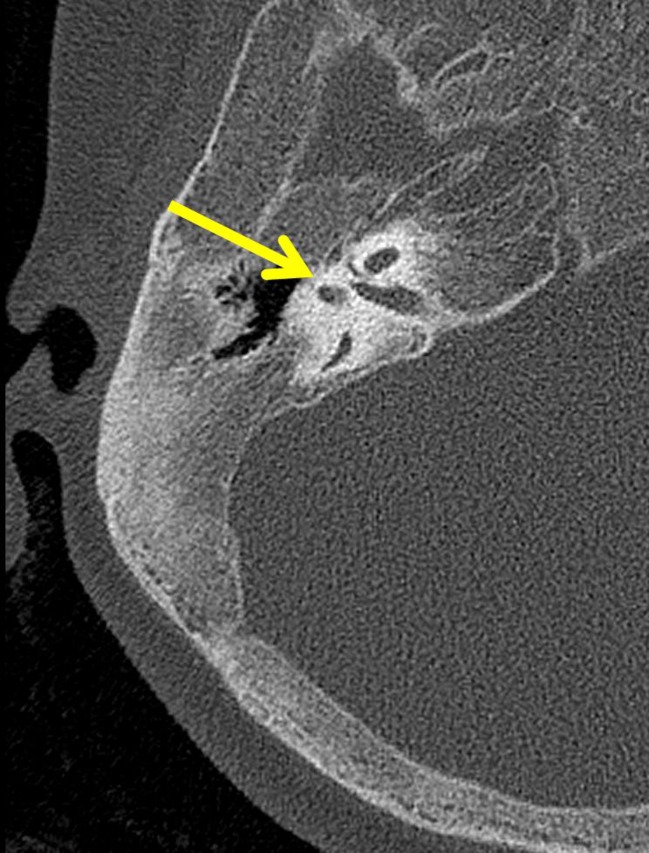
Diffuse hyperostosis of the bones of the skull. The facial canal is narrowed on the right (*arrow*)

A repeat brain MRI revealed significant bone abnormalities, with thickening of skull vault and skeletal skull base, but normal intracranial appearances. In view of these findings, a brain computed tomography (CT) scan was obtained (Fig. 1) and the findings were consistent with a grossly abnormal skull base and a narrowed right facial canal, which led to the right facial nerve palsy. The visualized bones showed generalized expansion, with cancellous bone demonstrating ground glass appearance. In addition, there was abnormal pneumatization of the paranasal and mastoid air cells, with just a few small pneumatized foci in the mastoids, and there was no pneumatization of the sphenoid or maxillary antra. The clivus was expanded and it was abnormal.

**Fig. 2:**
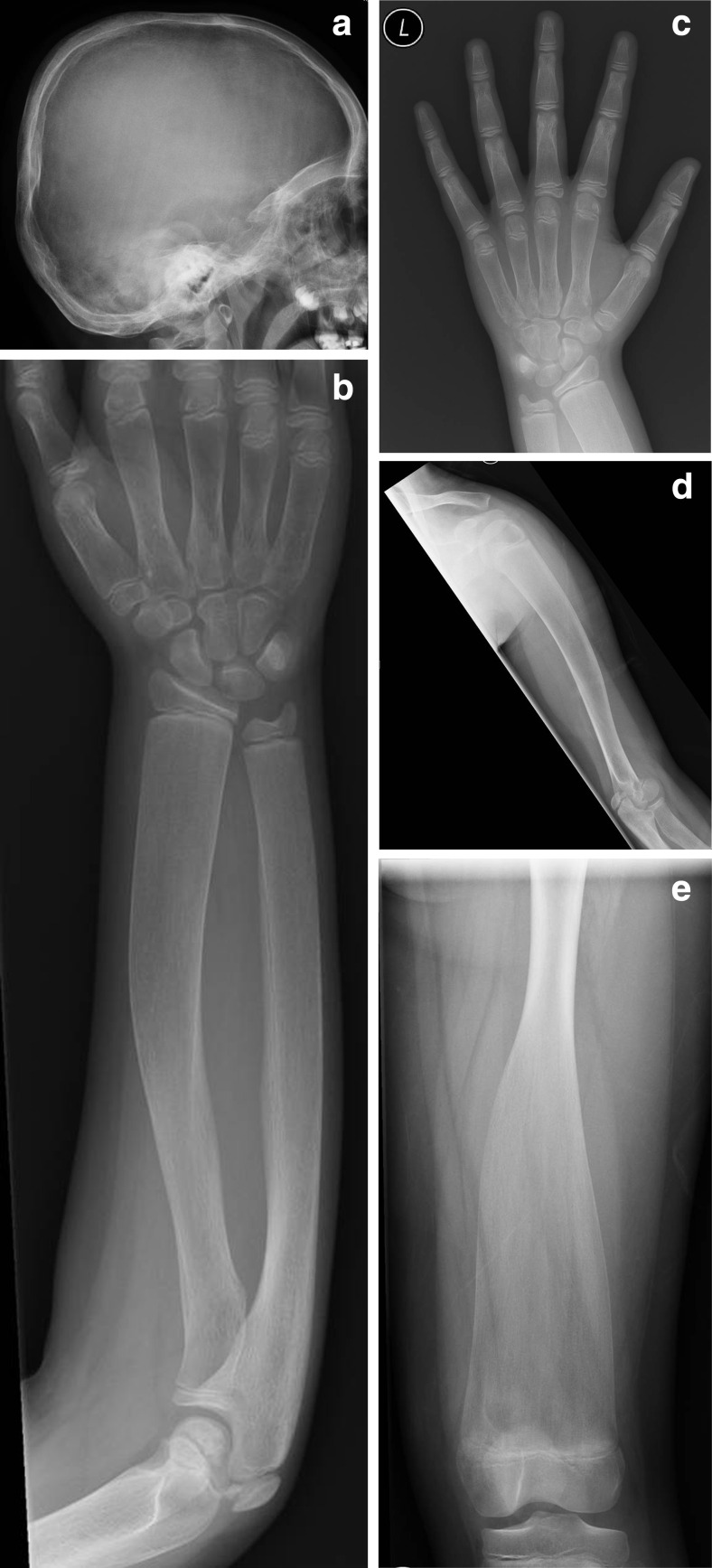
**a**–**e** There is thickening of the calvarium and mid-skull base sclerosis. The long bones are under modeled. Erlenmayer flask deformity of the femora and distal tapering of the humeri is noted. The tubular bones of the hand are under tabulated. There is distal broadening of the radius

A skeletal survey was also performed, which revealed abnormal thickening of the skull base and calvarium, dental malocclusion and mild deformities of the long bones with abnormal tubulation (Fig. 2a). There was widening of the lesser diaphyseal region and bowing of the humeri and radial bones (Fig. 2b). Widening of the metacarpals and phalanges (Fig. 2c) and also widening of the metatarsals and phalanges of both feet, of the clavicles, and of the ribs were also noted. The vertebral bodies were normal in appearance. Typical Erlenmeyer flask deformities were noted to both femurs with grossly widened metaphyses of long bones and marked cortical thinning and osteopenia (Fig. 2d, e). Based on the above radiological findings, the diagnosis of Pyle’s disease with facial nerve compression leading to chronic facial nerve palsy was set.

## Discussion

Pyle disease is a rare autosomal recessive disease with impressive and characteristic radiological findings but relatively mild clinical features. The clinically asymptomatic heterozygote may have minor disturbances of modeling of the tubular bones [5]. The condition is due to a defect in metaphyseal remodeling that results in gross widening of the metaphyses of long bones, marked thinning of the cortex, and osteoporosis [6]. The characteristic appearance of the long bones is described as Erlenmeyer flask deformity (EFD) and is usually more prominent in distal femur and proximal tibia. Spinal and jaw involvement have been described, while dental malocclusion is also a consistent finding [7, 8]. Patients with Pyle disease are often asymptomatic although genu valgus deformity may be a feature [9]. Dental anomalies may require orthodontic interventions, while skeletal anomalies may need orthopedic surgery [8].

The diagnosis of Pyle disease is usually made on the basis of clinical and radiological features, although the clinical signs are usually subtle and the diagnosis is made incidentally. The differential diagnosis of metaphyseal dysplasia from craniometaphyseal dysplasia is difficult, but important for the prognosis [10]. Table 1 shows the clinical and radiological characteristics of craniometaphyseal disease (autosomal dominant and recessive) and Pyle’s disease. In particular, Pyle disease has been confused mainly with the autosomal dominant form of craniometaphyseal dysplasia, which is milder than the recessive type [9]. Pyle disease differs from craniometaphyseal dysplasia clinically by the absence of any dysmorphic facial feature, milder skull involvement, lack of cranial nerve compression, and more striking long bone modeling defects leading to typical EFD [11, 12]. Our patient had no facial dysmorphic features, while she had a typical Erlenmeyer flask deformity in the long bones. The interesting finding is that this patient had an intense thickening of the skull and the calvarium, which led to narrowing of the right facial canal and to the compression of the facial nerve. Cranial nerve compression in Pyle’s disease has never been previously reported, since the limited cases described so far in the literature had either none or mild involvement of the skull.

**Table 1:** Craniometaphyseal dysplasias vs Pyle’s disease

	CMD AR type	CMD AD type	Pyle disease
Dysmorphic features	++++	++	–
Cranial sclerosis	+++	++	+
Cranial nerve palsy	++	+	–
Dental malocclusion	+	+	+
Bone fragility	–	–	+
Modeling defects in long bones	+	+	++
Erlenmayer flask	+	+	+
Club-shaped configuration	+	+	–

*CMD AR type* craniometaphyseal dysplasia autosomal recessive type, *CMD AD type* craniometaphyseal dysplasia autosomal dominant type

As far as the management is concerned, surgical reconstruction of the facial nerve could have been an option [4]. However, as there was such a significant improvement surgical intervention was not deemed to be appropriate. Dental malocclusion was another significant problem for our patient and teeth extractions were necessary. Otherwise, she is able-bodied and lives a normal life.

Chronic facial nerve palsy due to compression of the facial nerve in a patient with Pyle’s disease represents an unusual novelty, with a strong clinical impact, especially regarding the management and the prognosis. Furthermore, this case delineates the clinical spectrum and phenotype of such a rare clinical entity.
